# A survey from Turkey and Iran on comparison of risk factors and etiology in ischemic stroke

**Published:** 2019-10-07

**Authors:** Gulcin Benbir Senel, Ayse Deniz Elmali, Kaveh Mehrvar, Mehdi Farhoudi, Mohammad Aboutalebi, Mahsa Rezaei, Birsen Ince

**Affiliations:** 1 Department of Neurology, Cerrahpasa Medical Faculty, Istanbul University, Istanbul, Turkey; 2 Neurosciences Research Center, Tabriz University of Medical Sciences, Tabriz, Iran

**Keywords:** Cerebrovascular Stroke, Risk Factors, Etiology, Turkey, Iran

Although various advancements have been made to control incidence of stroke, the overall incidence, and the rate of morbidity of stroke, still increase in developing countries.^1^^-^^3^ In last years, the epidemiologic studies on ischemic stroke have been widely increased in Turkey and Iran.^1^^-^^3^ These two countries are located in similar geography with a similar historical background, though the environmental factors and lifestyle of general population in these two neighboring countries show peculiar differences. In this study, we aimed to make a comparison between data from Turkey and from Iran, in terms of etiologies and risk factors of ischemic stroke, to reveal region-related similarities or country-related differences.

We reviewed the files of 2534 patients with ischemic stroke followed up for 15 years in our Cerebrovascular Outpatient Clinics in Faculty of Neurology, Istanbul University (Cerrahpasa), Istanbul, Turkey. Data collection in Iran was made from 2314 patients with ischemic stroke followed up for 6 years in Stroke Unit, Neurosciences Research Center, Tabriz University of Medical Sciences, Tabriz, Iran. In the etiological classification, both centers used the Trial of ORG 10172 in Acute Stroke Treatment (TOAST) criteria^4^ upon clinical data, neurological examinations findings, and neuroimaging characteristics. Risk factors were recorded in detail, the statistical analyses were performed with the SPSS software (version 21, IBM Corporation, Armonk, NY, USA). Statistical tests used in the analysis were as χ^2^ or Fisher’s test for independent categorical variables, Student’s t test for normally distributed independent numerical variables, and Mann-Whitney U for not normally distributed independent numerical variables. The effects of covariates and independent variables were investigated by logistic regression. 

A P-value of > 0.050 was considered statistically significant. On the basis of TOAST classification, the biggest part was formed by the patients with undetermined in both groups etiology (Table 1). Almost one third of Turkish patients with ischemic stroke and one out of two Iranian patients had undetermined etiology (P < 0.001). Unknown etiology included patients with incomplete evaluation (about 3-10 percent of study group), those with negative evaluation in spite of detailed tests (about 50-80 percent of study group), and the patients with multiple causes (about 15-25 percent of study group). Among determined etiologies, it was observed that the most common underlying etiology of ischemic stroke was cardiac embolism in both groups of patients (P < 0.001). The second most common etiology of ischemic stroke was observed as small vessel disease in Turkish patients, versus large-vessel atherothrombosis in Iranian patients. In comparison, cardioembolism, small vessel diseases, and large vessel atherothrombosis were all more common in Turkish population, while other etiologies including dissection and vasculitis were more commonly observed in Iranian population (P < 0.001) (Table 1).

In the analysis of risk factors, the mean age of Turkish patients was calculated as 61.7 ± 13.4 years, which was 68.3 ± 13.8 years in Iranian patients; Turkish patients with ischemic stroke were significantly younger than Iranian patients (P < 0.001). Most of the patients were men among Turkish patients, while women constituted the majority of study population among Iranian patients. Other population-based studies from Iran have also reported higher rates of women in ischemic stroke.^1^^,^^3^ The most common risk factor was hypertension in both groups (P = 0.595), which was previously reported in separate studies from Turkey and Iran.^1^^-^^3^ The second most common risk factor in Turkish population was smoking, followed by the cardiac diseases (including coronary artery diseases, atrial fibrillation, valvular diseases, and patent foramen ovale). The second most common risk factor in Iranian population was history of cardiac diseases, followed by diabetes mellitus. In comparison between two populations, smoking, cardiac diseases, hyperlipidemia, and alcohol consumption were all more common in Turkish patients (P < 0.001) (Table 1). Smoking and alcohol consumption were observed as low in Iranian patients as the habitual risk factors. However, an intriguing feature was detected as very low incidence of hyperlipidemia in Iranian patients with ischemic stroke, which was reported three times higher in Turkish patients.

Building a healthy preventive strategy following stroke, identification of risk factors constitutes the most important step, and then comes to treat or to take under control these risk factors. World Health Statistics reported in year 2015 that different regions and cultures had different lifestyles and different vascular risk factors, and emphasized that every nation should document the characteristics of stroke and related features in their own population.^5^ Here, we observed that the etiology of ischemic stroke could not be detected in most of the patients both in Turkish and Iranian populations. 

**Table 1:** Etiology and risk factors of ischemic stroke in Turkish and Iranian patients.

**Variables**		**Istanbul (n = 2534)**	**Tabriz (n = 2314)**	**P**
Etiology (%)	Atherothrombosis	18.9	10.8	< 0.001
	Cardioembolism	22.8	16.6	< 0.001
	Small vessel diseases	20.3	3.6	< 0.001
	Other etiologies	4.9	10.5	< 0.001
	Unknown etiology	33.1	58.4	< 0.001
Age (year) (mean ± SD)		61.7 ± 13.4	68.3 ± 13.8	< 0.001
Gender (men, %)		53.9	48.6	< 0.001
Hypertension (%)		69.4	70.1	0.595
Diabetes mellitus (%)		26.7	25.0	0.168
Hyperlipidemia (%)		31.2	11.4	< 0.001
Cardiac diseases (%)		33.7	27.6	< 0.001
Smoking (%)		41.0	12.7	< 0.001
Alcohol (%)		18.0	< 5.0*	< 0.001

SD: Standard deviation

* Roughly estimated.

The most common underlying etiology of ischemic stroke was similar between two neighboring country, as cardiac embolism. The second most common etiology of ischemic stroke, however, was different between two groups, being small vessel disease in Turkish population and large-vessel atherothrombosis in Iranian population. Our results emphasize the importance of the need for national documentation in every country to better delineate the risk factors of ischemic stroke, hence it would be possible to decrease the incidence of stroke recurrences.

## Notes:

**How to cite this article:** Senel GB, Elmali AD, Mehrvar K, Farhoudi M, Aboutalebi M, Rezaei M, et al. A survey from Turkey and Iran on comparison of risk factors and etiology in ischemic stroke. Iran J Neurol 2019; 18(4): 176-8.
